# Effects of sublingual immunotherapy for dust mite on Th 17 / Treg cells in children with asthma

**DOI:** 10.1186/s13223-026-01010-8

**Published:** 2026-02-02

**Authors:** Fang Li, Xue Wang, Li Yin, Xiao Chu, Kun Wang, Wei Wang

**Affiliations:** 1 Department of pediatrics, The Second Hospital of Hebei Medical University, Shijiazhuang, China; 2 Department of pediatrics, Hebei Medical University Third Hospital, Shijiazhuang, China

**Keywords:** Dust mit, Sublingual immunotherapy, Th17 cell, Treg cell, Children

## Abstract

**Objective:**

observing the clinical effects of dust mite sublingual immunotherapy (SLIT) on children with asthma, changes of Th17 / Treg cells and related cytokines in order to investigate the possible pathological mechanism of immune tolerance induced by SLIT.

**Methods:**

Sixty children with asthma allergic to dust mites were included, divided into SLIT group (*n* = 30) and non-SLIT group (*n* = 30). Clinical symptoms of asthma in each group had been scored before, 1 year and 2 years after treatment. Meanwhile we also evaluated the proportion of Th17 and CD4^+^CD25^+^ Treg cells in peripheral blood using flow cytometry. Besides, cell culture supernatant was collected to detect the changes of IL-6, IL-10 and IL-17 levels.

**Results:**

We found that in SLIT group, asthma symptom and drug use score, Th17 cell percentages, IL-6 and IL-17 levels have significantly decreased throughout the study period (*p* < 0.05), while FEV1%, Treg cell percentages and IL-10 level have prominently increased throughout the study period (*p* < 0.05). By contrast, in non-SLIT group, asthma symptom score, lung function, Th17 cell percentages, IL-6 and IL-17 levels have all significantly improved, but on the whole lower than SLIT group (*p* < 0.05). However, we have observed no statistical differences in drug use score, Treg cell percentages, IL-10 level for non-SLIT group throughout the study period.

**Conclusions:**

SLIT of Dust mite drops could change T immune cell profiles whereas improve asthma symptoms. SLIT might reverse the functional imbalance of Th 17 / Treg cells and induce immune tolerance by upregulating Treg cell function and downregulating Th17 cell function.

## Introduction

Allergen immunotherapy (AIT) is the only method that can shorten the course of allergic disease [1]. Currently, studies of AIT mechanism have been focusing on immune tolerance [2]. However, the mechanism of how AIT induces the developing immune tolerance in T lymphocytes is not fully understood.

Th17 / Treg cells are a pair of T lymphocytes with pro-inflammatory / anti-inflammatory effects who participate in maintaining the dynamic balance of the body’s immune function [3]. The role of Th17 / Treg cells is also increasingly appreciated in asthma whose functional imbalance may lead to the occurrence and further development of asthma [4]. Our previous study has discovered the dysfunction of Treg cell in children who have bronchial asthma allergic to house dust mites, while the interesting point is that AIT can upregulate its function to exert therapeutic effects, yet the mechanism of this process has not been further examined [5].

We also have proved the presence of the vast majority of eosinophil accumulation in children with bronchial asthma allergic to dust mites and an imbalance of Th17 / Treg cell function in these children. Whether AIT can induce immune tolerance in children with bronchial asthma by reversing the Th17 / Treg cell function imbalance is unknown. The role of Th17/Treg cells is also increasingly appreciated in asthma, which is a hot issue in current research. There is no report about the changes of Th17 / Treg cells and their related cytokines in bronchial asthma children allergic to dust mite allergy after SLIT treatment over 2 years. Therefore, in this study, we aim to observe the effects of dust mite SLIT on children with asthma and changes of Th17 / Treg cells percentages as well as related cytokines to investigate the possible pathological mechanism of immune tolerance induced by SLIT.

## Patients and methods

### Patients

Children with mild and moderate non-acute onset bronchial asthma were finally selected for the study. Each child would need to meet the following inclusion criteria: (1) diagnosed with mild to moderate allergic asthma according to diagnostic criteria for bronchial asthma(GINA 2015) [6]; (2) allergic to dust mite diagnosed by skin prick test, wheal range ≥ ++, specific IgE ≥ 0.7Ku/L; (3) if child was also allergic to other allergens, such as spring and autumn pollen, cat, dog and animal fur or *Alternaria*, non-dust mite allergens must be <++ identified by skin prick test; (4) no other cardiovascular diseases, autoimmune diseases, or other underlying diseases. (5) None of the children had used systemic corticosteroids and other immunosuppressants, nor had they used antihistamines. The exclusion criteria: (1) diagnosed with chronic lung diseases, such as bronchopulmonary dysplasia, pulmonary fibrosis, congenital abnormal lung development and so on; (2) diagnosed with an immunodeficiency disease; (3) diagnosed with other serious illnesses; (4) diagnosed with severe bronchial asthma; (5) diagnosed with contraindications to pediatric pulmonary function tests. They were randomly divided into SLIT and non-SLIT groups according to the random number table method. Guardians of all children had provided the informed consent. Meanwhile, this study was approved by the Ethics Committee of the Second Hospital of HeBei Medical University.

### Treatment administration method

Patients were treated with conventional treatment for asthma or plus SLIT over 2 years. The treatment process consisted of a dose-escalation phase and a maintenance phase for 2 years. They were evaluated before the treatment and at the end of each year. The SLIT group was given sublingual dust mite drops (Zhejiang Wolwo Biological Technology Co., Ltd, Deqing, Zhejiang Province, China) numbered 1 to 4. No. 1, 2, and 3 which would be administered in weeks 1, 2, and 3 individually, with daily doses of 1, 2, 3, 4, 6, 8, and 10 drops. From the 4th week, the No. 4 would be given 3 drops once a day until the end of 2-year-treatment. The drops are swallowed after sublingual administration for 1 ~ 3 min at night at same time under the care of parents, and oral food intake was only allowed for 15 min after each administration until the end of the course of treatment.

All children treated with no systemic corticoids, other immunosuppressants and no anti-allergic drugs within 4 weeks before blood collection. Both groups had the same basic medication, and were given with small to moderate doses of inhaled corticosteroids (Budesonide inhalant), without combining long-acting antihistamines and antileukotriene drugs.

### Reagents and equipment

The fluorescein-isothiocyanate (FITC) mouse anti-human CD4, mouse anti-human (PE Alexa Fluor 700), Alexa Fluor 647 anti-human IL-10, APC anti-human IL-17, PE anti-human Foxp3, anti-human CD25, human IL-6, human IL-10, human IL-17 ELISA kits were all purchased from eBioscience, Inc (San Diego, CA, USA). Cytofix/Cytoperm solution, lymphocyte separation medium and RPMI-1640 medium were bought from Gibco (USA). The flow cytometer (FACSCantoII) was purchased from BD Biosciences (New Jersey, USA); Automatic allergen detector (Uni- CAP100) was purchased from Pharmacia (Uppsala, Sweden); the CO_2_ thermostatic incubator(MCO-175) was purchased from SANYO (Osaka, Japan); Fully automatic microplate reader (Multiskan MK3) was purchased from Thermo Fisher Labsystems (USA).

### Standard for clinical asthma symptom score and asthma drug use score

Evaluation was performed with night and morning asthma symptom scores [7]. The night symptoms would be scored from 0 to 4 points as following: “0” for no symptoms; “1” for early or one arousal during sleep; “2” for early and more than two arousals during sleep; “3” for many arousals during sleep; “4” for no sleep at night. Daytime symptoms were scored as following: “0” for no symptoms; “1” for a little symptoms and short duration; “2” for more than twice very short symptoms; “3” for mild symptoms occurred at more times but with less affects to study and life; “4” for more time and more symptoms with affects on the study and life; “5” for the symptoms are serious and the children cannot live and study normally. The higher score indicated more severe asthma symptoms.

Asthma drug use score own a total score of 7 points as below in which these following drugs could be used in combination according to the child’s condition. “1” for the β2 receptor agonists or leukotriene receptor antagonists, individually; “2” for inhaled corticosteroids; “3” for inhalational glucocorticoids corticosteroids (ICS) combined with a long-acting β2 receptor agonist (LABA).

### Cell isolation and culture

The fasting peripheral venous blood (5 mL) would be drawn from each subject before treatment, 1 year and 2 years of treatment. The blood would be placed in sterile heparin tubes separated by Ficoll density-gradient centrifugation to isolate the PBMCs. Then cells were supposed to be washed with Hank’s solution for three times and resuspend in RPMI-1640 medium with 10% fetal bovine serum (FBS). The PBMCs would be then stimulated with dust mite leaching solution at dose of 20 µg/mL for 48 h. The cell culture supernatant would be obtained to evaluate IL-6, IL-10 and IL-17 levels by ELISA, and cells would be collected to analyze the Th17 cells (CD4^+^ IL-17^+^ T cells) and Treg cells (CD4^+^ CD25^+^ FOXP3^+^ IL-10^+^ T cells) percentages.

### Statistical analysis

Data is analyzed using the SPSS24.0 software. All sets of measurement data are expressed as mean ± standard deviation ($\bar{x}$± S). The Chi-Square test ($\chi$^2^ test) is used for the comparison of categorical data. For the comparison between groups (SLIT and non-SLIT groups), t-test is performed to compare data with normally distributed variance alignment, while the unpaired Mann-Whitney U test is used for non-normal distribution or uneven variance, and paired multi-sample Friedman-test is employed for within-group (pre-treatment, 1 year of treatment, 2 years of treatment) comparisons. *P* < 0.05 is considered as statistically significant.

## Results

### Subject characteristics

**Fig. 1:**
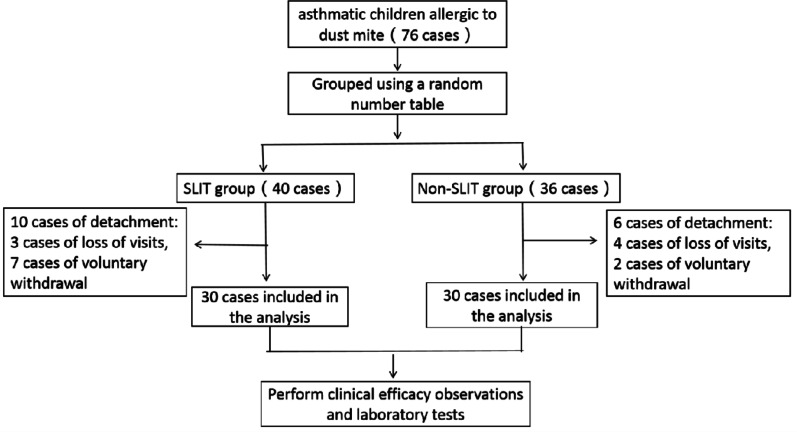
CONSORT flowchart. CONSORT, consolidated standards of reporting trials

A total of 76 children with bronchial asthma were initially included in this study, but 6 cases were lost in the non-SLIT group, of whom 4 were lost to follow-up and 2 voluntarily withdrew from the study for personal reasons. The 10 cases were lost in the SLIT group, of whom 3 were lost to follow-up and 7 voluntarily withdrew from the study for personal reasons. Finally, 60 cases were included and randomly divided into SLIT and non-SLIT groups with 30 patients in each group. In the SLIT group, 19 boys and 11 girls were aged 9.22 ± 2.64 years. In the non-SLIT group, 18 boys and 12 girls were aged 8.90 ± 2.44 years. There were no differences in the two groups in age, sex, severity, duration, lung function, presence, associated disease, dust mite specific IgE level, and positive allergen distribution of the skin prick test results (See Fig.1, Tables 1 and 2).

**Table 1:** General information of children

	SLIT group	Non-SLIT group	t / $\chi$^2^ value	*P* value
Cases	30	30		
Gender (Male/ Female)	19/11	18/12	0.071 ($\chi$^2^)	0.791
Age (Year)	9.22 ± 2.64	8.90 ± 2.44	0.482 (t)	0.631
Disease course (Year)	4.73 ± 1.19	4.10 ± 1.99	− 1.357 (t)	0.098
Dust mite specificity IgE (KU/mL)※	134.65 ± 142.52	138.70 ± 106.64	− 0.99 (t)	0.322
Family history（N/Y）	16/14	19/11	0.617 ($\chi$^2^)	0.432
Rhinallergosis （N/Y）	9/21	11/19	0.300 ($\chi$^2^)	0.584
Allergic dermatitis（N/Y）	19/11	21/9	0.300 ($\chi$^2^)	0.584

Data are expressed as the mean ± standard deviation, ※* P* < 0.05, was considered to be statistically significant difference

**Table 2:** Distribution of allergen skin prick test results in the children

	SLIT group	Non-SLIT group	$\chi$^2^ value	*P* value
Cases	30	30		
Family history (N/Y)	16/14	19/11	0.617	0.432
Rhinallergosis (N/Y)	9/21	11/19	0.300	0.584
Allergic dermatitis (N/Y)	19/11	21/9	0.300	0.584
House dust (N/Y)	25/5	24/6	0.111	0.739
Mould Ⅱ (N/Y)	23/7	26/4	1.002	0.317
Alternaria (N/Y)	25/5	26/4	0.131	0.718
Dog hair (N/Y)	22/8	23/7	0.089	0.766
Cat epithelium (N/Y)	20/10	23/7	0.739	0.390
Weed pollen (N/Y)	20/10	17/13	0.635	0.426
Ragweed (N/Y)	17/13	15/15	0.268	0.605
Artemisia argyi (N/Y)	16/14	18/12	0.271	0.602
Hay dust (N/Y)	26/4	25/5	0.131	0.718

※* P* < 0.05, was considered to be statistically significant difference

### Asthma symptom scores

**Fig. 2:**
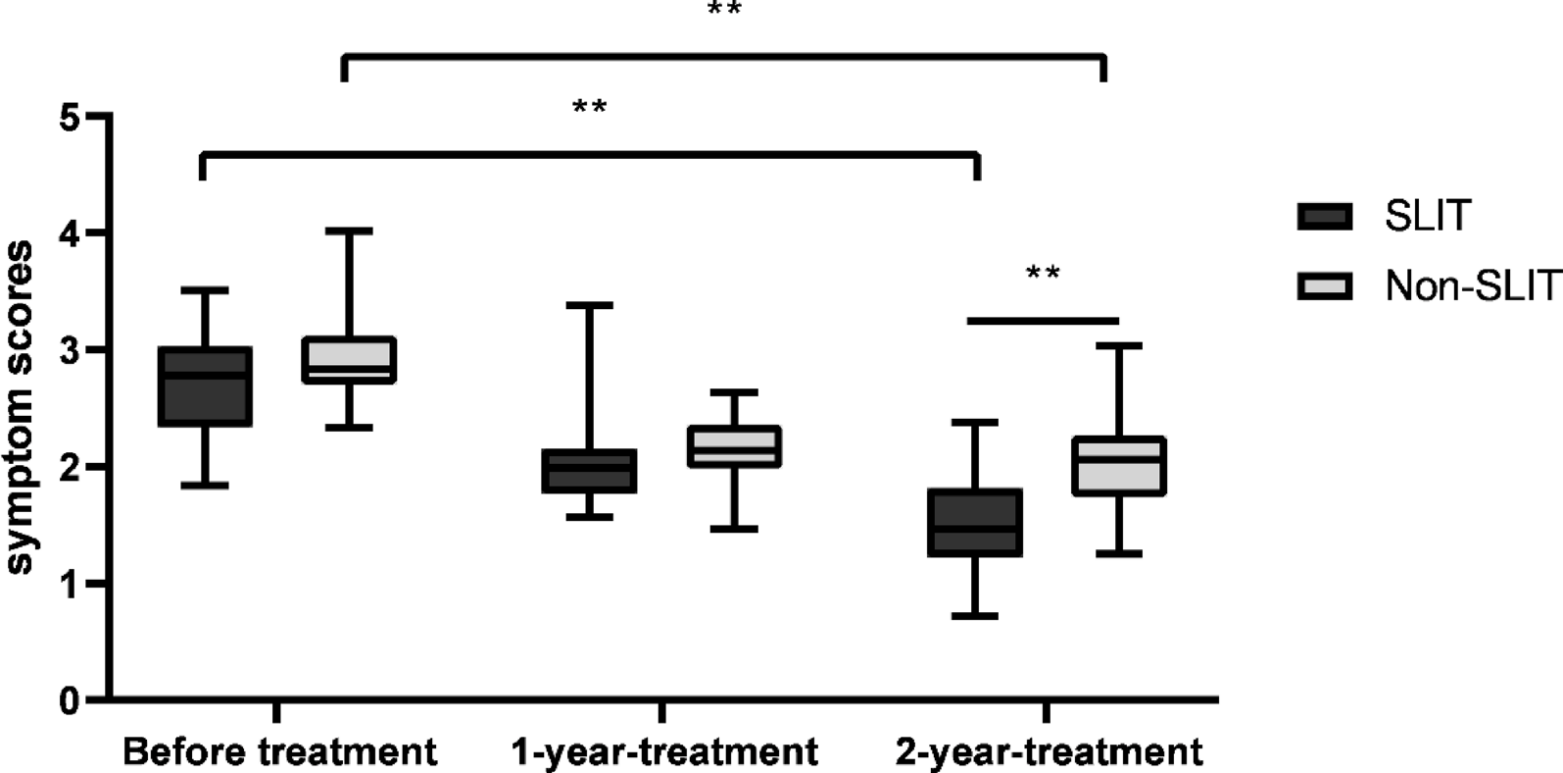
Asthma symptom scores in SLIT group and non-SLIT group. Mean asthma symptom scores obviously decreased in SLIT group over the study period (F = 50.334,* p* < 0.01); symptom scores did not differ within the non-SLIT group until treatment after 2 year compared with before treatment (F = 39.800,* p* < 0.01). **p < 0.01

Mean asthma symptom scores in the SLIT group were respectively 2.72 ± 0.45, 2.12 ± 0.45 and 1.52 ± 0.46 in before treatment, 1 year and 2 years after treatment. In addition, the scores obviously decreased throughout the research period (F = 50.334, *p* < 0.01). Although the mean asthma symptom scores have shown the decline in the non-SLIT group, we found no statistically different changes until treatment after 2 year compared with before treatment (F = 39.800, *p* < 0.01). Intergroup comparison has revealed that the range ability of asthma symptoms scores in the SLIT group was remarkably higher than that of non-SLIT group (*p* < 0.05). See Fig. 2.

### Asthma drug use scores

**Fig. 3:**
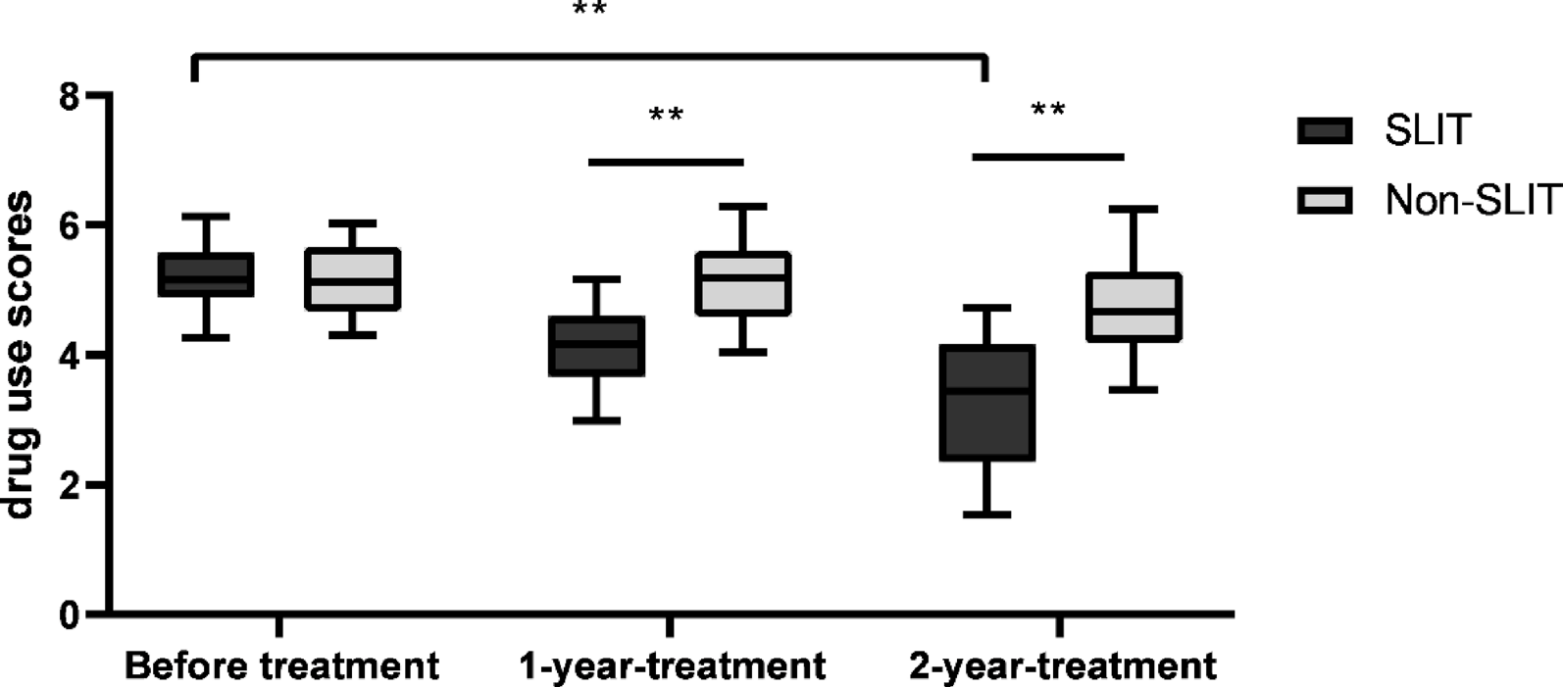
Drug scores in SLIT group and non-SLIT group. Mean drug scores obviously decreased in SLIT group over the study period (F = 60.000,* p* < 0.01); drug scores did not differ within the non-SLIT group (F = 4.467,* p* = 0.107). **p < 0.01

Mean asthma drug use scores in the SLIT group were respectively 5.18 ± 0.47, 4.13 ± 0.62 and 3.27 ± 1.02 in before treatment, 1 year, 2 years after treatment. And the scores significantly declined throughout the research period (F = 60.000, *p* < 0.01). The mean drug use scores appeared to decline in the non-SLIT group, but the changes had no statistically significant differences (F = 4.467, *p* = 0.107). Intergroup comparison has revealed that the change amplitude in the SLIT group was significantly greater than non-SLIT group (*p* < 0.01). See Fig. 3.

### Lung function

**Fig. 4:**
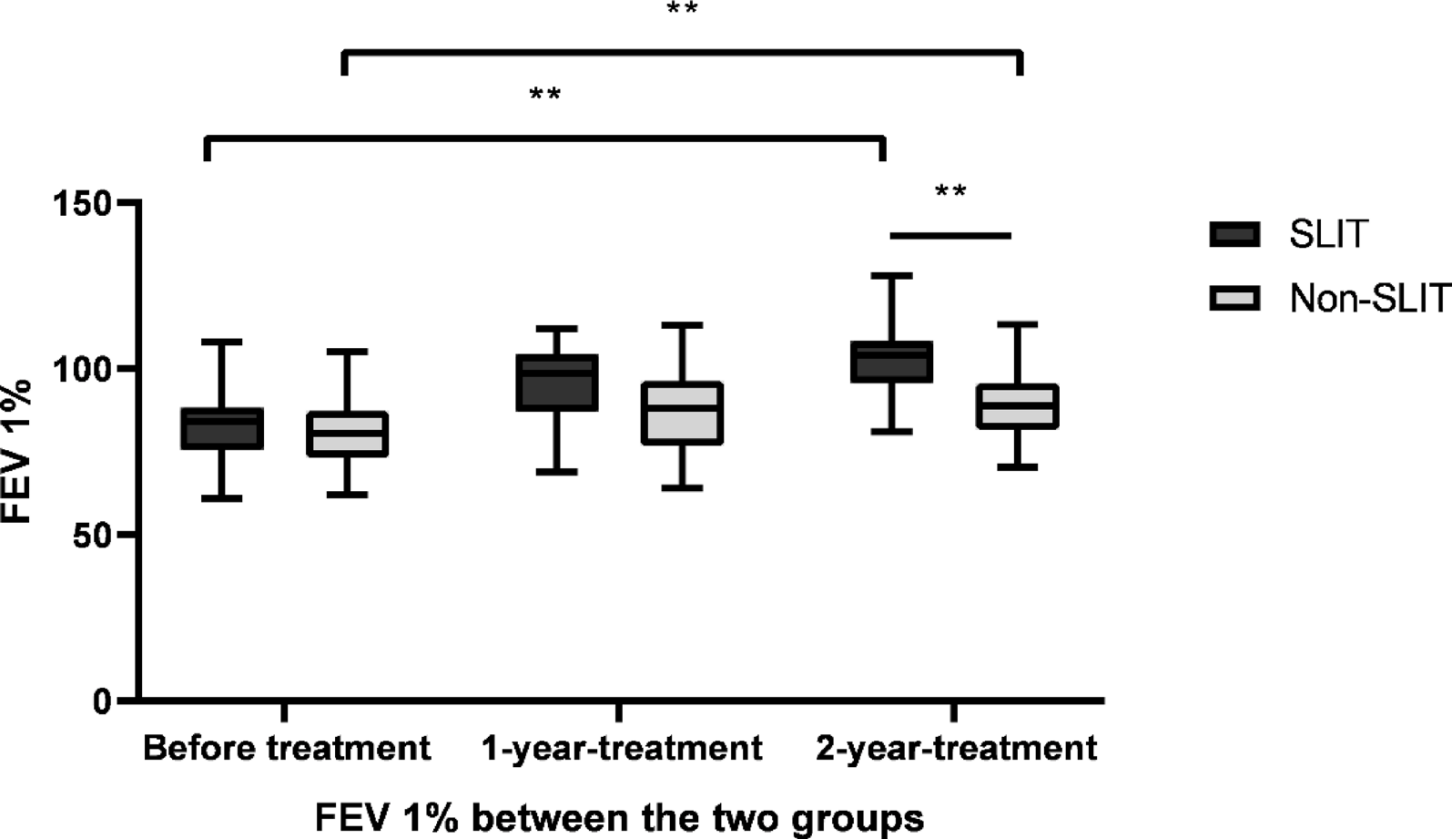
FEV 1% in SLIT group and non-SLIT group. Mean FEV 1% significantly increased over the treatment period in SLIT group (F=34.889,* p* < 0.01). And it also increased significantly in the non-SLIT group after 2 years of drug treatment compared with before treatment (F = 27.042,* p* < 0.01). **p < 0.01

Mean FEV 1% in the SLIT group were 82.83 ± 12.17%, 95.47 ± 11.68% and 102.83 ± 12.17%, before treatment, and 1 year, 2 years after treatment, respectively. And FEV 1% significantly increased over the treatment period (F = 34.889, *p* < 0.01). FEV 1% increased significantly in the non-SLIT group after 2 years of drug treatment compared with before treatment (F = 27.042, *p* < 0.01). It has shown that the change amplitude was significantly higher in the SLIT group than non-SLIT group by intergroup comparison (*p* < 0.01). See Fig. 4.

### Decreasing of Th17 cells percentages after treatment

**Fig. 5:**
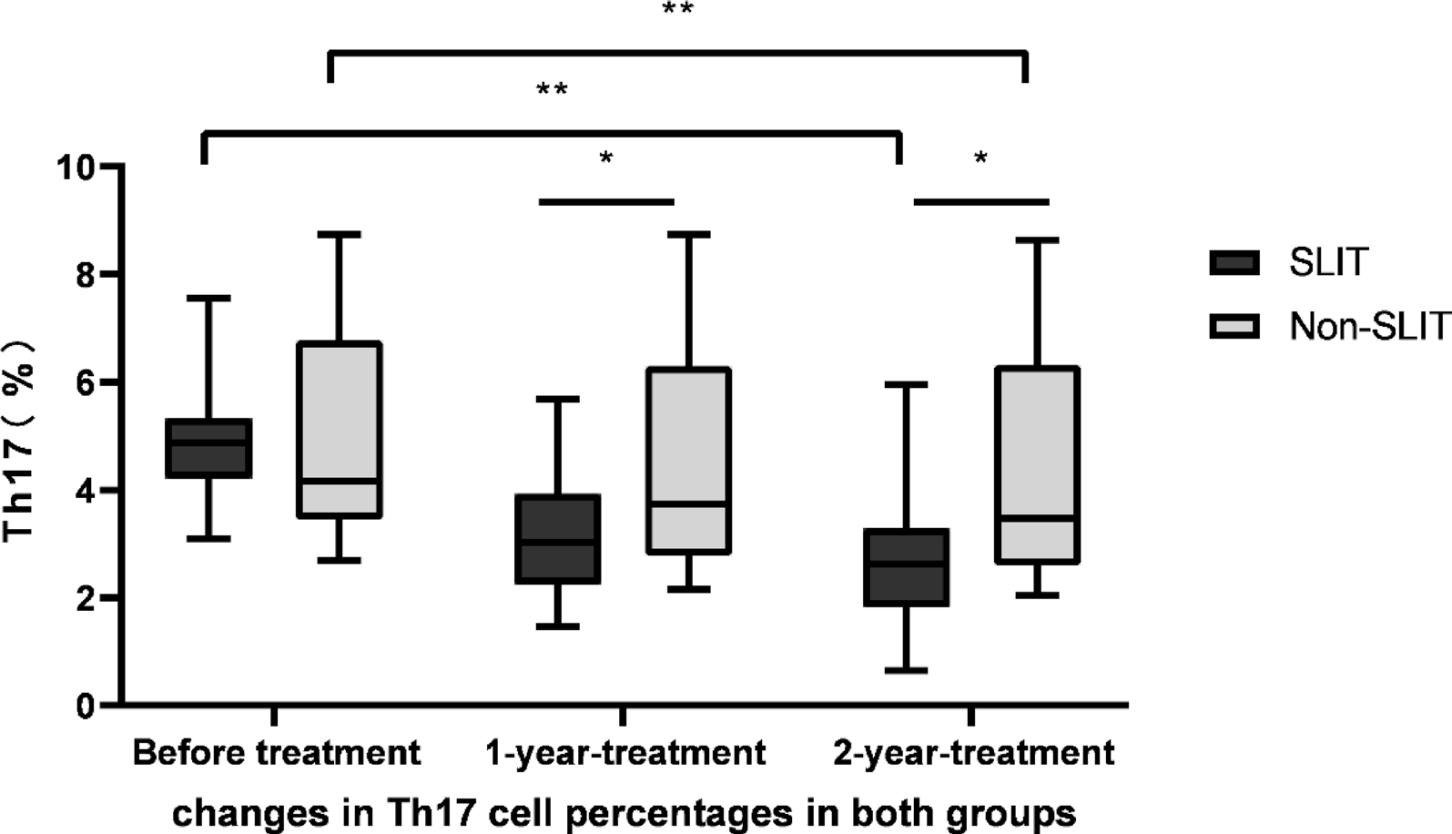
Decreases in Th17 cells following treatment. FACS was used to detect IL-17-posititive PBMCs in patients of SLIT group and non-SLIT group. Th17 cell percentage significantly declined throughout the research period in SLIT group (F = 47.400,* p* < 0.01); Th17 cell also gradually declined throughout the study period in non-SLIT group (F = 44.826,* p* < 0.01).While the magnitude of change was obviously higher in the SLIT group than that of the non-SLIT group (*p* < 0.05). *p < 0.05, **p < 0.01

To determine the effects of SLIT on the Th17 cells proportion in PBMCs, the FACs analysis was conducted to identify and quantify CD4 and cells secreting IL-17. Percentages of Th17 cells in SLIT group were 4.90 ± 0.96, 3.15 ± 1.07 and 2.68 ± 1.16 before treatment, 1 year and 2 years after treatment, individually. Th17 cell percentage gradually declined throughout the research period in SLIT group, with significantly differences (F = 47.400, *p* < 0.01). The percentages of Th17 cells were 4.96 ± 1.87, 4.53 ± 2.07, 4.41 ± 2.12 before treatment, 1 year and 2 year after treatment in non-SLIT group, individually. Before treatment, there was no statistically significant difference in Th17 (CD4⁺IL-17 A⁺) cell levels in PBMCs stimulated with house dust mite extract between the SLIT and non-SLIT groups (*P* > 0.05).The percentage of Th17 cell also gradually declined throughout the study period in non-SLIT group (F = 44.826, *p* < 0.01). While the magnitude of change after treatment was obviously higher in the SLIT group than that of the non-SLIT group (*p* < 0.05). See Fig. 5.

### Increase in the Treg cell percentages after treatment

**Fig. 6:**
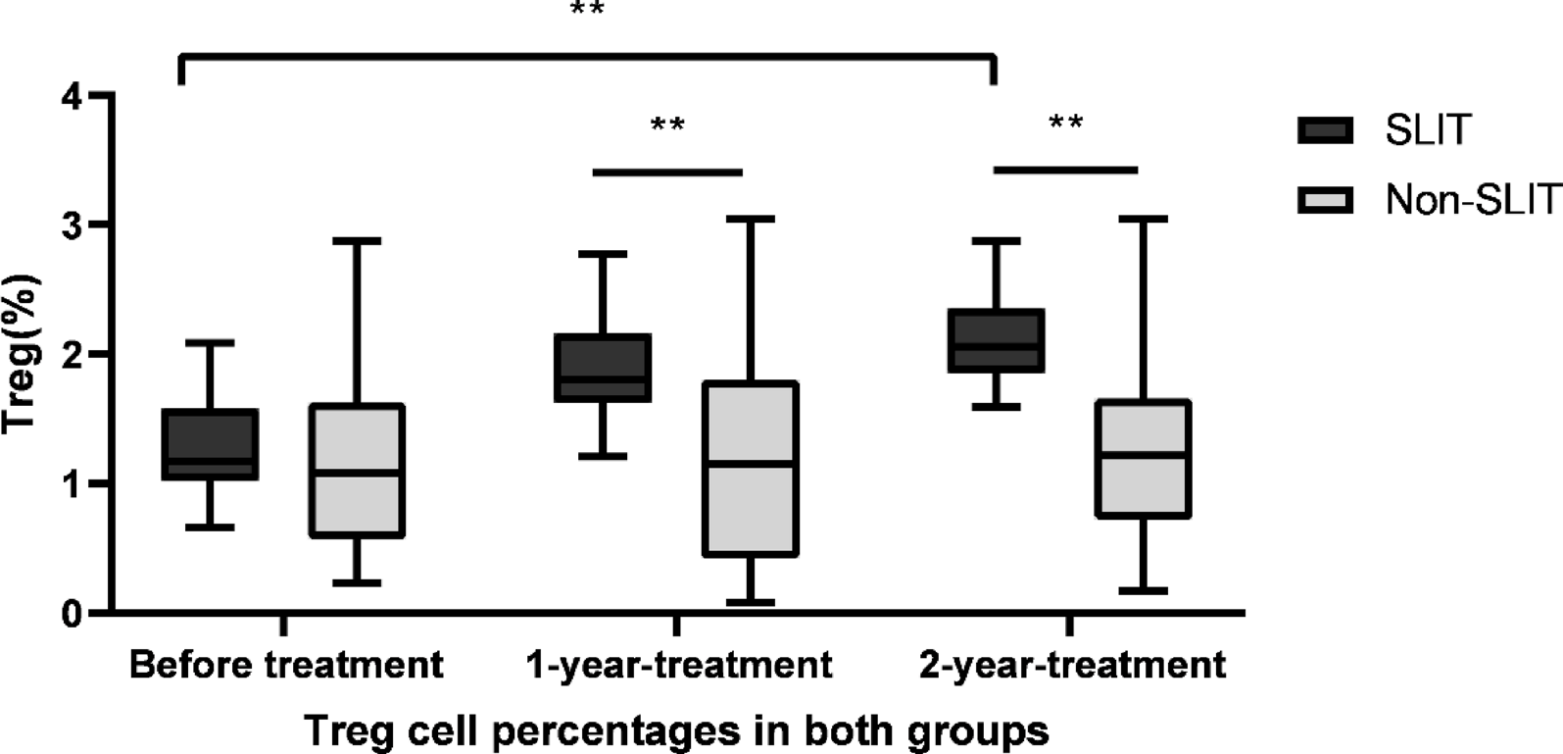
Increases in Treg cells following treatment. FACS was used to detect CD4+CD25+Foxp3+IL-10+ T cells in patients of SLIT group and non-SLIT group. Treg cell percentage significantly increased throughout the research period in SLIT group (F = 40.267,* p* < 0.01); Treg cell were unchanged throughout the study period in non-SLIT group (F = 3.525,* p* = 0.172). **p < 0.01

To determine whether SLIT alters the proportion of Treg cells in PBMCs, we further identified and quantified CD4^+^CD25^+^Foxp3^+^IL-10^+^ T cells by FACs. Before treatment, there was no statistically significant difference between the SLIT and non-SLIT groups in the levels of IL-10–producing Treg cells (CD4⁺CD25⁺Foxp3⁺IL-10⁺) in PBMCs stimulated with house dust mite extract (*P* > 0.05).Its percentages significantly enhanced throughout the research period in SLIT group (F = 40.267, *p* < 0.01), from 1.27 ± 0.37 at baseline to 1.89 ± 0.38, 2.11 ± 0.35 at 1 year and 2 years after treatment. Although percentage of Treg cell appeared to increase in the non-SLIT group, no statistically significant differences were observed (F = 3.525, *p* = 0.172). The percentages of Treg cells in the treated SLIT group were significantly higher than non-SLIT group, with a statistically significant difference (*P* < 0.01). See Fig. 6.

### Changes of IL-6, IL-10 and IL-17

**Fig. 7:**
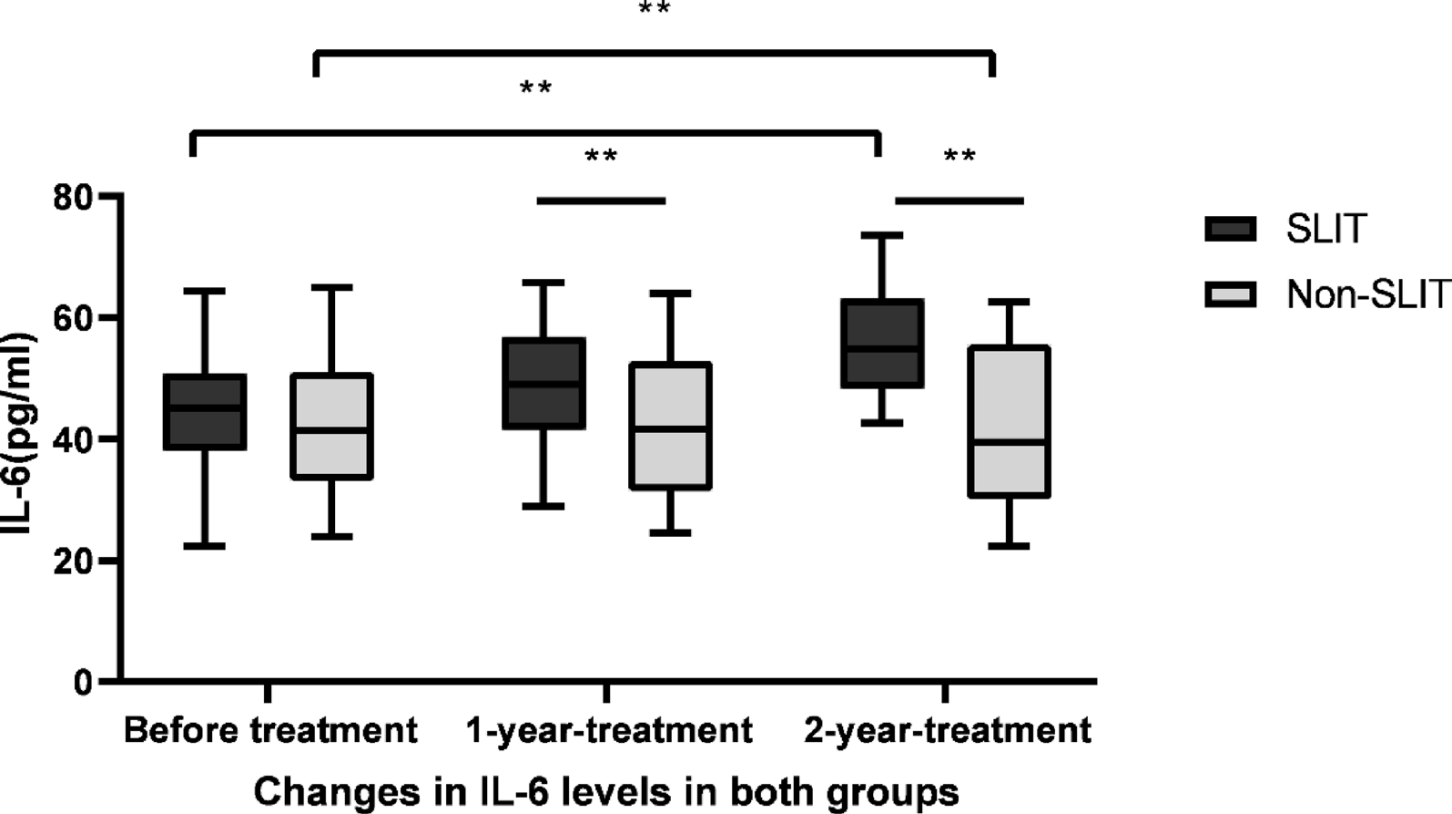
Decreases in IL-6 following treatment. ELISA was used to identify the IL-6 levels in supernatants of PBMCs in patients of SLIT group and non-SLIT group. IL-6 level significantly declined throughout the research period in SLIT group (F=50.400,* p* < 0.01); IL-6 also gradually declined throughout the study period in non-SLIT group (F = 24.267,* p* < 0.01). **p < 0.01

**Fig. 8:**
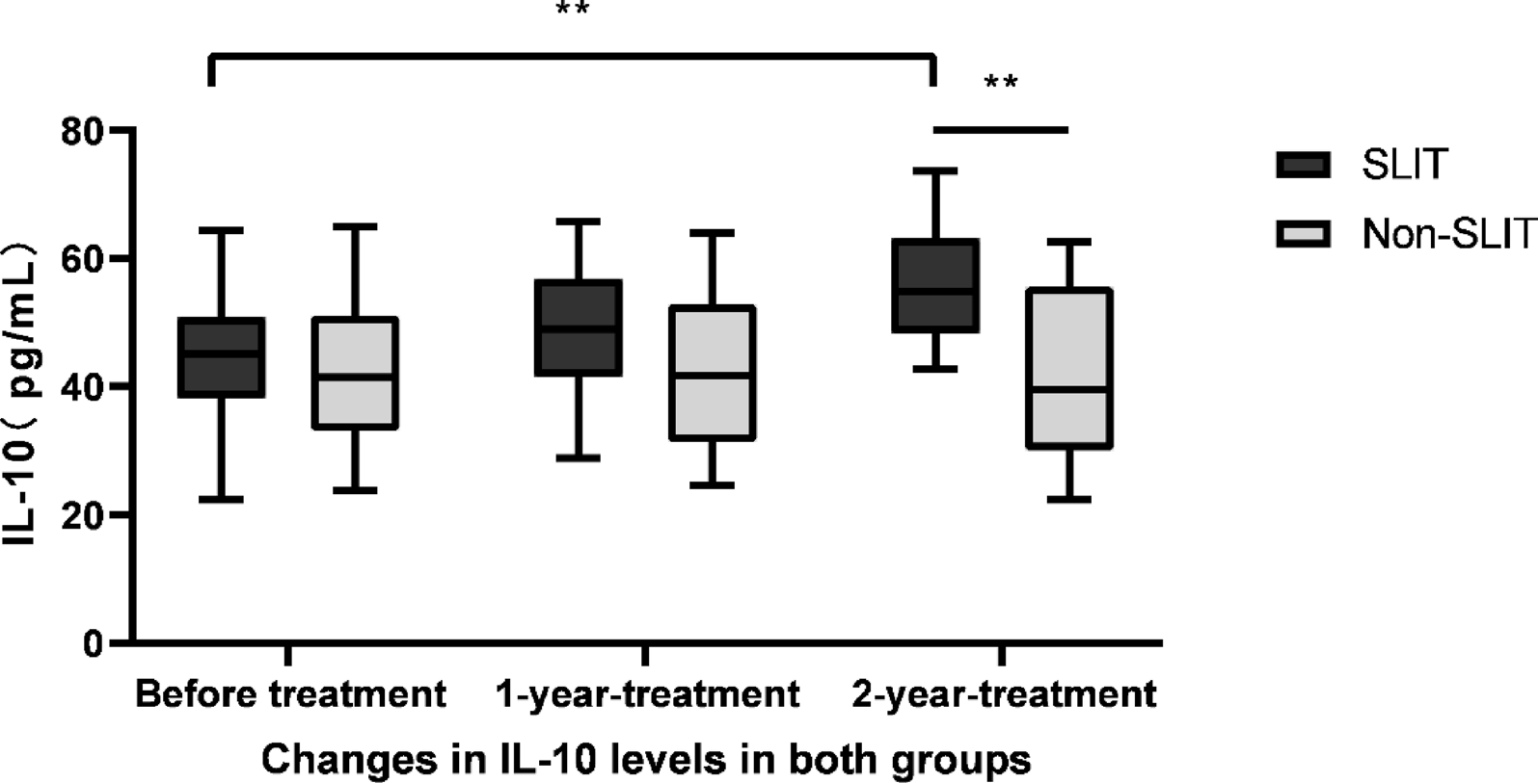
Increases in IL-10 following treatment. ELISA was used to identify the IL-10 levels in supernatants of PBMCs in patients of SLIT group and non-SLIT group. IL-10 level significantly increased throughout the research period in SLIT group (F = 49.043,* p* < 0.01); while IL-10 level was unchanged throughout the study period in non-SLIT group (F = 5.765,* p* = 0.056). **p < 0.01

**Fig. 9:**
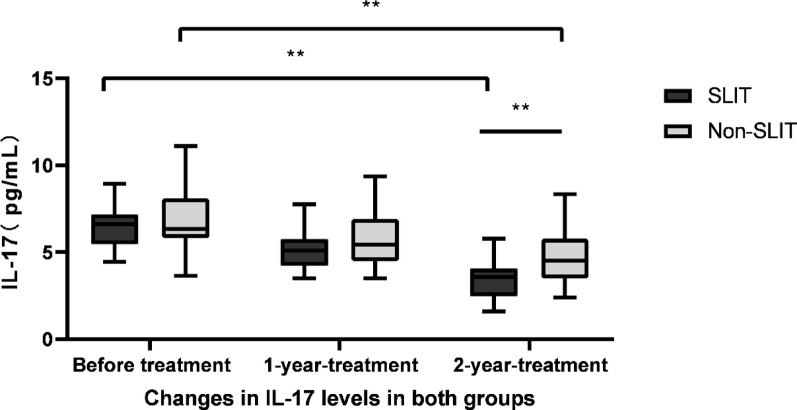
Decreases in IL-17 following treatment. ELISA was used to identify the IL-17 levels in supernatants of PBMCs in patients of SLIT group and non-SLIT group. IL-17 level significantly decreased throughout the research period in SLIT group (F = 59.051,* p* < 0.01); IL-17 level also gradually declined throughout the study period in non-SLIT group (F = 38.000,* p* < 0.01). **p < 0.01

The ELISA was conducted to identify the IL-6, IL-10 and IL-17 levels in supernatants of PBMCs. IL-6 levels in SLIT group were 55.02 ± 9.16, 45.44 ± 8.18 and 36.62 ± 6.81 pg/mL before treatment, 1 year and 2 years after treatment, respectively. It gradually declined in SLIT group over the study period, with significantly differences (F = 50.400, *p* < 0.01, Fig. 7). In contrast to IL-6, IL-10 levels were significantly increased throughout the study period in SLIT group (F = 49.043, *p* < 0.01), which were 44.05 ± 9.41, 48.84 ± 9.21 and 56.12 ± 8.15 pg/mL before treatment, 1 year and 2 years after treatment, respectively (Fig. 8). While IL-17 levels were 6.46 ± 1.15, 5.11 ± 1.08 and 3.41 ± 1.02 pg/mL before treatment, 1 year and 2 years after treatment in SLIT group, respectively, and the differences were statistically significant (F = 59.051, *p* < 0.01, Fig. 9). Although IL-6, IL-10 and IL-17 levels in non-SLIT group also appeared the similar tendency of changes, the magnitude of change was apparently greater in the SLIT group than that of non-SLIT group (all *p* < 0.01, Figs. 7, 8 and 9).

## Discussion

The current international consensus is that allergen immunotherapy (AIT) has definite curative effects in allergic rhinitis and allergic asthma. AIT is a therapeutic vaccination used to treat IgE-mediated allergies to common allergens such as pollen, house dust mites, and aspiration insect venom. AIT has two main forms in clinical use: subcutaneous immunotherapy (SCIT) and sublingual immunotherapy (SLIT). SCIT is the classic form of administration, but the need for subcutaneous administration increases the pain and the risk of serious systemic adverse reactions. However, SLIT is easy to take, and rarely causes serious systemic adverse effects, so it attracts more and more attentions and likely to replace SCIT [8].

The mechanism of how specific immunotherapy induced immune tolerance in T lymphocytes is not fully understood. It is believed that the main mechanism of SLIT induction of immune tolerance is to promote the conversion of antigen-sensitized CD4^+^ Th0 cells into Treg cells and Th1 cells and thus inhibit the Th2 cell-associated immune response [9]. Dust mite drop immunotherapy is a kind of sublingual specific immunotherapy. It is made of dust mite antigen active protein, which can induce the body to generate specific antibodies, then combine with allergens, thus blocking the allergen and basophils and mast cells interaction, block the occurrence of type I allergic reaction, at the same time can inhibit T lymphocytes and eosinophils gathered in the target organ, inhibit degranulation changes in lymphocytes and exert regulatory effects on the immune system [10].SLIT can induce immunoglobulin class switching in B cells, shifting antibody production from IgE to IgG, particularly the IgG4 subclass. SLIT has been shown to increase serum levels of allergen-specific IgG subclasses, especially IgG1 and IgG4, by 10- to 100-fold.These IgG antibodies, often referred to as “blocking antibodies,” can not only prevent allergen–IgE interactions and inhibit the cross-linking of FcεRI on effector cells and antigen-presenting cells, thereby reducing IgE-dependent histamine release, but also suppress delayed allergen-specific T-cell responses by inhibiting the binding of allergen–IgE immune complexes to low-affinity IgE receptors on antigen-presenting cells, particularly B cells.

Th 17 / Treg cells play an important role in maintaining the dynamic balance of immune function in vivo. Under some specific conditions, Treg cells and Th 17 cells can interconvert, such as changes in cytokine concentration [11]. Th17 cells play an important role in the immunopathogenesis of various autoimmune diseases and tumors. IL-17 is mainly a cytokine secreted by Th 17 cells, and it cannot replicate the asthma model if IL-17 is knocked out. IL-17 is mainly involved in severe asthma and hormone-resistant asthma, which is dominated by neutrophil infiltration [12].

IL-6 is involved in the proliferation and differentiation process of Th17 cells. It has a key regulatory role in the differentiation of initial CD4^+^ T cells into Th17 cells and Treg cells [13]. Binding of IL-6 to cell surface receptors can trigger the lung JAK / STAT3 signaling, thus playing a key regulatory role in Th17 / Treg differentiation [14]. The activated JAK kinase phosphorylates STAT3, p-STAT3 dimerizes and transplaces into the nucleus to initiate expression of downstream related genes such as RORγt, IL-17 A / F, prompting differentiation of initial CD4^+^ T lymphocytes to Th17. Meanwhile, STAT3 can also promote the methylation of the CpG island of the enhancer upstream of FOXP3 gene and inhibit its expression. SOCS1 and SOCS3 negatively regulate IL-6 / STAT3 signaling. Animal experiments found that downregulation of IL-6 in the respiratory tract achieved good results in treating asthmatic animals [15]. Clinical experiments found that the effect of inhaled corticosteroids in asthma was related with downregulation of IL-6 levels in serum [16].

Treg cells are a group of cells that exert a negative regulatory effect on the cellular immune function, among which the FOXP3 protein is its most important transcription factor. Treg cells mainly inhibit the T cell-mediated inflammation dominated by eosinophil infiltration in the respiratory tract, thus reducing the respiratory allergic reaction and preventing the development of asthma [17]. The Treg cells predominantly secrete IL-10 and TGF-β. IL-10 may have an immune dual effect, which promotes the development of inflammatory responses rather than anti-inflammatory under some conditions, and its dual function is not only dependent on the target cells, but also on the concentration of IL-10 [18]. IL-10 can affect the function of T cells at different stages of inflammation. Moreover, IL-10 was also able to inhibit the differentiated in Th 17 cells [19].

The imbalance of Th17 / Treg cell function plays an important role in the development of bronchial asthma, Hu Y et al. reported that differentiation of Th 17 / Treg cells is implicated in the pathogenesis of allergic asthma [20]. They found a significant Th 17 / Treg imbalance in the child with bronchial asthma, in which the percentage of Th 17 cells was significantly increased while the percentage of Treg cells was significantly reduced, and the IL-17 levels were significantly increased while the IL-10 levels were significantly reduced. However, Katrien Van der Borgh et al. treated the mouse model of bronchial asthma allergy with house dust mite (HDM) with prophylactic SLIT treatment, and they found the SLIT treatment could increase the levels of treg and Th 17 in the lungs of the asthma model mice [21]. Thus, the role of Th 17 / Treg in SLIT is inconclusive, and it is unclear whether SLIT can play a role in immune tolerance by reversing the imbalance of Th17 / Treg cell function. Therefore, we aimed to preliminarily explore the mechanism of SLIT by observing the changes in Th17 / Treg cells and corresponding cytokine levels in bronchial asthma children allergic to dust mite receiving SLIT. Our study found that asthma symptom score and drug use score Th17 percentages, IL-6 and IL-17 levels were significantly decreased in SLIT group throughout the study period (*p* < 0.05). In contrast, FEV1%, Treg cell percentages and IL-10 level significantly enhanced in SLIT group throughout the study period (*p* < 0.05). Asthma symptom score, lung function, Th17 cell percentages, IL-6 and IL-17 levels all significantly improved in non-SLIT group even all lower than SLIT group (*p* < 0.05). But no statistical difference was observed in drug use score, Treg cell percentages and IL-10 level for non-SLIT group. Our study found that Treg levels remained at a high level after 2 years of sublingual specific immunotherapy, which might be related to the child still in desensitization treatment. It was reported that the optimal treatment time of SLIT was 3 years, and it was considered that it can maintain better long-term efficacy after 3 years of drug withdrawal [22]. The 2-year SLIT course did not maintain the long-term effect. We will further explore the changes of Th 17 / Treg cells and their associated cytokines levels in children after the cessation of treatment to evaluate the long-term efficacy of specific immunotherapy.

The results showed that Th17 / Treg cell function imbalance existed in children with bronchial asthma allergic to eosinophil accumulation, especially Treg cell dysfunction. Although drug treatment can improve the clinical symptoms and the level of some cytokines in children, it cannot reduce the use of asthma drugs and upregulate Treg cell function. SLIT can upregulate Treg cell function and downregulate Th17 cell function to reverse the imbalance of Th17 / Treg cell function and induce immune tolerance. However, whether it reverses Th17 / Treg cell function by inhibiting IL-6 secretion needs further investigation.

## Conclusion

In conclusion, Our study proved that SLIT of Dust mite drops could alter T immune cell profiles and improves asthma symptoms. And SLIT might reverse the functional imbalance of Th 17 / Treg cells to induce immune tolerance by upregulating Treg cell function and downregulating Th17 cell function.

## Data Availability

All data supporting the findings of this study are available within the paper and its Supplementary Information.
